# Beyond the bedside: protocol for a scoping review exploring the experiences of non-practicing healthcare professionals within health professions education

**DOI:** 10.1186/s13643-023-02364-5

**Published:** 2023-11-09

**Authors:** Helen R. Church, Megan E. L. Brown, Lynelle Govender, Deborah Clark

**Affiliations:** 1 Faculty of Medicine and Health Sciences, University of Nottingham, Medical School, Queen’s Medical Centre, Nottingham, NG7 2UH UK; 2 School of Medicine, Newcastle University, Newcastle, UK; 3 Division of Anatomical Pathology, Faculty of Health Sciences, University of Cape Town, Cape Town, South Africa; 4 Division of Clinical Medicine, School of Medicine and Population Health, Faculty of Health, University of Sheffield, Sheffield, UK

**Keywords:** MeSH terms, Health personnel, Health professions, Allied health personnel, Faculty, Medical, Faculty, Nursing, Health Educators, Non-MeSH terms, Non-practicing, Ex-clinician, Health professions education, Healthcare professional

## Abstract

**Background:**

The number of healthcare professionals leaving clinical practice and transitioning to alternative careers in health professions education is increasing. Among these non-practicing healthcare professionals, concerns have been reported regarding tensions in relation to identity, role, and credibility in their new field. There are suggestions that this is a particularly pressing issue for minoritised professionals who make this transition. Support is critical to attract and retain diverse talent within health professions education teaching and research. The purpose of this scoping review is to explore the career experiences of non-practicing healthcare professionals who work in health professions education internationally.

**Methods:**

Arksey and O’Malley’s framework has been utilised to guide the design of this scoping review process and will be used throughout the course of the review. A comprehensive search of seven electronic databases and limited search of Google Scholar will be conducted, as well as a hand search of eligible article reference lists. Two reviewers will independently screen all articles based on inclusion criteria, with conflicts resolved by a third reviewer. Data from included articles will be charted, collated, and analysed thematically. Meta-data will be summarised quantitatively.

**Discussion:**

This scoping review aims to explore the role and experiences of non-practicing healthcare professionals working within health professions education. The review will follow established scoping review guidelines and will include studies from various regions and languages, provided an English translation is available. The study remit will be broad, including both quantitative and qualitative studies, as well as reviews and opinion papers. Limitations may include the exclusion of non-English articles and potential difficulty of identifying papers which discuss the experiences of non-practicing clinicians. However, the review will provide insight into the current knowledge on what it is like to be a non-practicing clinician working within health professions education and identify gaps for both future research, and future support for those making this career transition.

**Systematic review registration:**

Open Science Framework Registration https://doi.org/10.17605/OSF.IO/485Z3

**Supplementary Information:**

The online version contains supplementary material available at 10.1186/s13643-023-02364-5.

## Background

The number of doctors, nurses and other healthcare professionals leaving clinical practice is growing [1], and, simultaneously, interest in alternative careers in adjacent disciplines, such as pharmaceuticals, research, and education are growing [2]. The Covid-19 pandemic, and resultant strain on the healthcare workforce in terms of workload and wellbeing, is exacerbating what was an already emerging trend [3]. Though health professions education (HPE; inclusive of both teaching and educational research) represents an attractive change in career for many leaving clinical practice, the experiences of individuals making this transition and remaining in HPE are currently unknown. Understanding this career path (which is increasing in popularity) is critical, so that appropriate support for those making this transition can be offered, and so that HPE can attract and retain diverse talent.

HPE faculty is currently populated by those from both clinical and non-clinical backgrounds. Most usually, faculty have a strong sense of belonging to the community associated with their primary training/academic background (e.g., medical, nursing, dentistry if clinical; or biomedical, psychology, or education if non-clinical). It is common within HPE for clinicians to hold joint clinical and academic roles. For clinicians who are no longer practicing (I.e., they have a clinical background, but no longer provide direct patient-facing health or social care), issues of identity can arise [4]. Professional identity can be defined as how people perceive themselves, and how others perceive them in the context of their professional role. For non-practicing clinicians in HPE, the act of leaving clinical practice can make navigating roles in HPE, and HPE identities particularly challenging [4]. Difficulties with identity development and lack of belonging can have many negative impacts, including: low levels of confidence; challenges in establishing and maintaining professional relationships and networks; higher levels of stress and burnout; and job dissatisfaction [5, 6].

Concerningly, there is emerging evidence that those who are no longer practicing are more likely to be minoritised (e.g., women, racialised people, people who act as primary caregivers) [7–9] and so this issue also has important impacts on diversity and equity within HPE. Though there is emerging research on those who are non-practicing clinicians within HPE, and research more broadly on those who leave healthcare professions, there are no scoping or systematic reviews on this topic. As such, little is known about the experiences of those who have transitioned from clinical practice to roles within HPE. Further, there is a lack of a cross-professional view – there may be commonalties (or differences) in experiences for non-practicing clinicians in HPE with different primary professional backgrounds, and a more interprofessional approach is necessary is explore this further. Our research collaborative is interested in this phenomenon – the journey and professional/career experiences of ex-clinicians who provide valuable educational services across the HPE disciplines.

The objective of this scoping review is to assess the extent and nature of literature which documents the experiences of non-practicing healthcare professionals (across professionals, and internationally) within HPE. Specifically, this review will focus on the journey and experience of non-practicing clinicians who provide valuable educational services in any academic or clinical setting, e.g., a higher education institution (e.g., lecturer), or in a healthcare setting (trainer in clinical practice). across the HPE disciplines. By identifying and analysing the existing literature, this scoping review will contribute to a better understanding of the challenges and opportunities facing non-practicing healthcare professionals in HPE and provide insights into how to support them in their roles.

## Methods

### Review question

The process of creating the research question for this scoping review involved several steps. First, we conducted a preliminary review of the literature to gain an understanding of the existing research. Based on this initial review, we identified a gap in the literature regarding the career experiences of non-practicing professionals across professions and internationally. We then engaged in a series of discussions to refine the research question and ensure that it was clear, focussed, and relevant to our research goals. Our research question, which provides a framework for our scoping review, is as follows:

*“What is known about the career experiences of non-practicing healthcare professionals (where non-practicing is defined as individuals with clinical backgrounds who no longer have clinical roles directly relating to patient care) across professions, and internationally, in health professions education?”*

### Methodological framework

Prospero and Epistemonikos were searched to establish that no similar literature reviews had been registered. Arksey and O’Malley’s [10] scoping review methodology will be used to guide this project. We have selected this methodology to add rigour to our review and ensure that the process of our review is transparent and replicable.

This scoping review has been registered with the Open Science Framework (ID: https://doi.org/10.17605/OSF.IO/485Z3). We registered the review on this platform to receive input and feedback from the wider academic community and to prevent duplication of research effort.

This review will be reported using the Preferred Reporting Items for Systematic Reviews and Meta-Analyses (PRISMA) guidelines, drawing on the extension for scoping reviews specifically (PRISMA-ScR) [11]. This protocol has been structured and complied using the guidance provided by the PRISMA extension for protocols (PRISMA-P) [12] (see the PRISMA-P checklist in Additional file 1).

### Eligibility criteria

The eligibility criteria for this scoping review are based on participants, concept, context, and types of sources. This study aims to analyse literature focussed on non-practicing healthcare professionals from the disciplines of medicine, nursing, dentistry, and allied health professions who now work in education and no longer practice clinically. The review will consider articles from any country or region, so long as an English translation of the article can be sourced. We are unable to include non-English language articles, given resource constraints. The types of sources that will be included in this review are all types of empirical study design (e.g., experimental and quasi-experimental study designs, analytical and descriptive observational study designs, qualitative studies), systematic reviews, and commentary and opinion papers. We will also search the grey literature. The review aims to capture any data pertaining to the career experiences of non-practicing healthcare professionals across professions and internationally in health professions education. A full list of our eligibility criteria is provided in Table 1, below.

**Table 1:** Eligibility criteria

Eligibility criteria	Description
Participants	Subjects of included studies should be non-practicing healthcare professionals within the following disciplines: • Medicine • Nursing • Dentistry • Allied Health Professionals We will accept synonyms of “non-practicing”, including but not limited to: ex-practitioner, ex-clinician, and non-clinical (if there is also reference to a health professions background). We are hoping most included articles will define what they mean by “non-practicing”. If they do not, we will apply the definition given in our research question (*individuals with clinical backgrounds who no longer have clinical roles directly relating to patient care*) to assess whether a retrieved article meets this eligibility criteria
Concept	The concept to be explored is the career experiences of healthcare professionals who now work in education (including educational research/scholarship/leadership) and no longer practice clinically
Context	The review aims to capture any articles which contain data (empirical research or otherwise) pertaining to non-practicing clinicians. Articles from any country or region will be included, provided an English translation of the article can be sourced
Types of sources	This scoping review will consider all types of study design both qualitative and quantitative In addition, systematic reviews that meet the inclusion criteria will also be considered, depending on the research question Commentary and opinion papers will also be considered for inclusion in this scoping review Grey literature (e.g., conference abstracts, white papers) will be eligible for inclusion

### Search strategy

The search strategy will aim to locate both published and unpublished studies. An initial limited search of MEDLINE was undertaken to identify articles on the topic in January 2023. The text words contained in the titles and abstracts of relevant articles, and the index terms used to describe the articles were used to develop the full search strategy, with the assistance of a medical librarian. Our general search strategy can be found below in Table 2, and will be adapted to each database to optimise search outputs.

**Table 2:** Search strategy

**Search element**	**Population**		**Concept**		**Context**
Synonyms	Health Personnel doctor* OR nurse OR clinician* OR chiropodis OR podiatrist* OR dieticia* OR orthoptist OR radiographer OR paramedic OR physiotherapist OR osteopath OR pharmacist OR optician OR chiropractor OR radiographer OR language therapist OR speech therapist OR pyschologist OR prosthetist OR occupational therapist OR operating department practitioner OR arts therapist OR biomedical scientist OR clinical scientist health worker OR health provider OR health professional OR health personnel	AND	Ex-practitioner OR career change OR career transition	AND	Education, Medical OR Health Education OR Vocational Education OR Education Professional OR Education OR Teaching OR Curriculum OR Educat* OR teach*

The search strategy, including all identified keywords and index terms, will be adapted for each included database and/or information source. The reference list of all included sources of evidence will be screened for additional studies. Studies published in the English language will be included. Study publication dates will not be limited.

The databases to be searched (from inception onwards) are:

- AMED (Allied and Complementary Medicine)
- CINAHL
- EMBASE
- ERIC
- PsychInfo
- Medline
- Scopus
- Google Scholar

Sources of unpublished studies / grey literature to be searched include:

- ProQuest dissertations
- OpenGrey archive

### Evidence selection

Following the search, all identified citations will be collated and uploaded into Covidence (https://www.covidence.org) and duplicates removed. Titles and abstracts will then be screened by two or more (a third reviewer will resolve any disagreements) independent reviewers for assessment against the inclusion criteria for the review. We will utilise this approach to ensure comprehensive identification of all relevant retrieved sources.

Potentially relevant sources will then be retrieved for full text review. If we cannot retrieve a full text of a paper (drawing on our various institutional accesses), we will contact the listed corresponding author of that paper by email or through other networking organisations (e.g., ResearchGate) to request the article. We will allow the contacted authors two weeks to respond with a full text. If, after this time, we are not provided with the article, the paper will be excluded based on inaccessibility.

The full text of successfully retrieved citations will be assessed in detail against the inclusion criteria by one reviewer. Reasons for exclusion of sources of evidence at full text that do not meet the inclusion criteria will be recorded and reported in the scoping review. Any uncertainties that arise at any stage of the selection process will be resolved by discussion with one or more co-reviewers. The results of the search and the study inclusion process will be reported in full in the final scoping review and presented in a Preferred Reporting Items for Systematic Reviews and Meta-analyses extension for scoping review (PRISMA-ScR) flow diagram [10, 13].

### Data extraction

Data will be extracted from all papers which are deemed eligible for inclusion following full text review by one reviewer. Data will be extracted using an a-priori draft extraction form (Additional file 2), which includes specific details on the participants included in each study, the study type and context, the focus (concept) of the study, and key findings (focussing on those which help us to answer our research question regarding the experiences of non-practicing healthcare professionals in HPE). The form was designed through review of our research question, and through discussion as a team.

We anticipate that this draft form will evolve as we review studies in our screening phases and that we may add or refine categories of the form. We have provided our draft data extraction form in the attached additional file (noting the proviso that this may be edited throughout the process of data extraction to capture all relevant and important insights). Any modifications that we make to our data extraction form will be documented in the final publication of this scoping review.

### Data analysis and presentation

Extracted data will be analysed and presented in two workstreams:

1. Demographics

Meta-data pertaining to the included articles will be presented in both tabular/numerical form as counts/frequencies with an accompanying narrative description. Variables such as year of publication, geographical origin of the article, population (which healthcare profession(s)) are central to the article will be explored. For articles communicating empirical research, additional data will be extracted pertaining to study design, methodology adopted and number of participants.

2) Thematic analysis

An iterative approach will be taken to develop the final themes discussed in relation to the review. Several categories will be decided prospectively prior to data extraction (deductive framework) as per the attached draft extraction form. However, as the authors of this review work through the retrieved articles, new categories are likely to be identified inductively. By the end of data extraction, all themes will be considered for every included article. These will be presented both using figures/diagrams (if the authors consider a visual representation useful following review of final retrieved data, and the synthesised analysis) to communicate the concepts and the relationships between them, and through narrative.

## Discussion

The scoping review presented in this proposal aims to explore the career experiences of non-practicing healthcare professionals in health professions education across various countries and disciplines. This review design adheres to the guidelines set by Arksey and O'Malley's framework and the PRISMA-P guidance to ensure systematic and rigorous methodology, and we will review these frameworks throughout the course of conducting the review. By combining quantitative meta-data analysis, and deductive and inductive thematic analysis, this review aims to evaluate the relevance of identified literature to explore what is known about non-practicing healthcare professionals working within health professions education.

Although we have designed a comprehensive literature search in collaboration with a trained information specialist, it is important to acknowledge the limitations of this review. The exclusion of non-English language articles may result in valuable international research being overlooked and limit our global perspective on this topic. Further, relevant studies that do not list their non-practicing context within their title and abstract may be missed – we have worked with a librarian to try and mitigate this in our search strategy and develop synonyms for non-practicing, including terms relating to career change or transition. This means it is likely we will need to screen a greater breadth of articles not relevant to our research question but should help us be more comprehensive and exhaustive in our search. Another potential limitation of this scoping review is the possibility of publication bias. It is possible that studies that reported negative findings may not have been published (especially if those findings, which may relate to negative career experiences, are perceived as reflecting badly on a local organisation or institution). This may affect the comprehensiveness of our review and skew our report towards a more positive synthesis of findings. We will be mindful of this possibility in our interpretation of the scoping review data, and exercise caution if we recognise a lack of negative experience representation. Despite these limitations, this review will provide a helpful overview of the current state of knowledge on non-practicing healthcare professionals, their role, and experiences in health professions education. Given no such synthesis exists, and the numbers of non-practicing healthcare professionals transitioning into the field are increasing, this scoping review is valuable and timely.

The findings of this scoping review will be disseminated through publication in a relevant peer-reviewed journal and presentation at relevant health professions education conferences. Additionally, the results of this review will be shared with health professions educators in each of our respective institutions to inform future research and the development of educational programmes that cater to non-practicing healthcare professionals.

In conclusion, this scoping review will contribute to the understanding of the career experiences of non-practicing healthcare professionals in health professions education. The review will highlight any gaps in current knowledge and provide insights into the experiences (both positive and negative) of non-practicing healthcare professionals working within health professions education. Ultimately, this review will help to inform recommendations for the development of educational programmes that better cater to the needs of non-practicing healthcare professionals transitioning to health professions education and inform an ongoing programme of research in this topic area, which may lead to improvements in healthcare education and delivery.

### Supplementary Information

**Additional file 1. **Completed PRISMA-P tool.

**Additional file 2. **Data extraction form draft.

## Data Availability

Not applicable.
