# Supplementary material for: Beyond the bedside: protocol for a scoping review exploring the experiences of non-practicing healthcare professionals within health professions education
Source: Syst Rev. 2023 Nov 9;12:207. doi: 10.1186/s13643-023-02364-5 (PMC10633985; doi:10.1186/s13643-023-02364-5)
Supplement: Supplementary file 1 — Additional file 1. Completed PRISMA-P tool. [file 13643_2023_2364_MOESM1_ESM.docx]

**PRISMA-P 2015 Checklist**

# **This checklist has been adapted for use with systematic review protocol submissions to BioMed Central journals from Table 3 in Moher D et al**:**** Preferred reporting items for systematic review and meta-analysis protocols (PRISMA-P) 2015 statement. *Systematic Reviews* 2015 ****4****:1

# An Editorial from the Editors-in-Chief of *Systematic Reviews* details why this checklist was adapted - **Moher D, Stewart L & Shekelle P**:**** Implementing PRISMA-P: recommendations for prospective authors. *Systematic Reviews* 2016 ****5****:15

| **Section/topic** | **#** | **Checklist item** | **Information reported** | | **Line number(s)** |
| --- | --- | --- | --- | --- | --- |
|  |  |  | **Yes** | **No** |  |
| **ADMINISTRATIVE INFORMATION** | | | | | |
| **Title** | | | | | |
| Identification | 1a | Identify the report as a protocol of a systematic review | 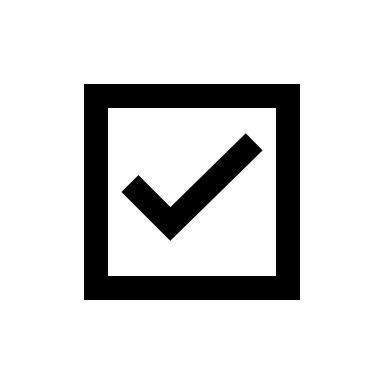 |  | Pg 1, line 1  Pg 4, line 17 |
| Update | 1b | If the protocol is for an update of a previous systematic review, identify as such |  | 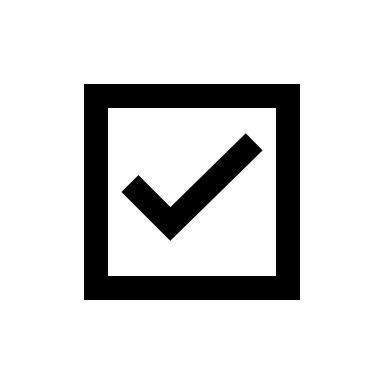 | Not applicable (not update) |
| **Registration** | 2 | If registered, provide the name of the registry (e.g., PROSPERO) and registration number in the Abstract | 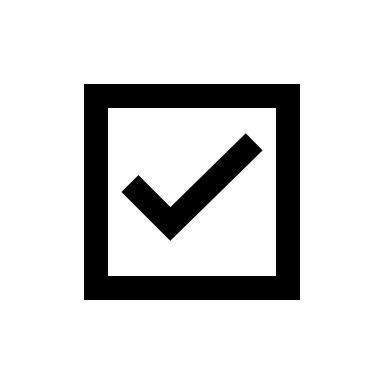 |  | Pg 2, lines 9-10 |
| **Authors** | | | | | |
| Contact | 3a | Provide name, institutional affiliation, and e-mail address of all protocol authors; provide physical mailing address of corresponding author | 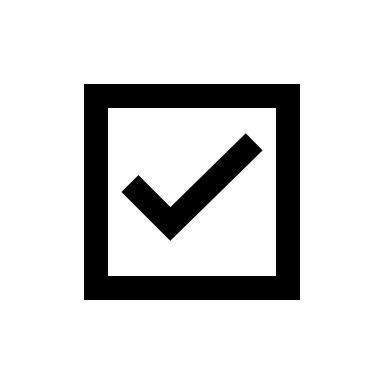 |  | Pg1, lines 5-14 |
| Contributions | 3b | Describe contributions of protocol authors and identify the guarantor of the review | 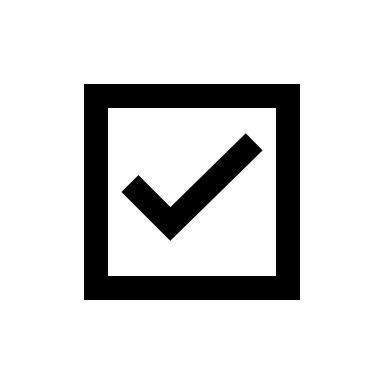 |  | Pg 11, lines 26-9 |
| **Amendments** | 4 | If the protocol represents an amendment of a previously completed or published protocol, identify as such and list changes; otherwise, state plan for documenting important protocol amendments |  | 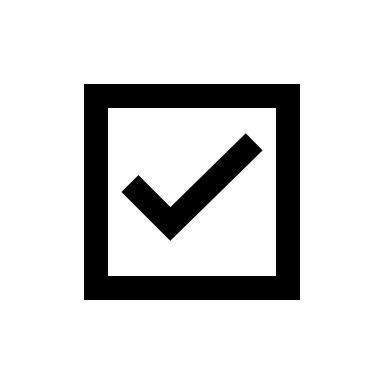 | Not applicable (no amendments) |
| **Support** | | | | | |
| Sources | 5a | Indicate sources of financial or other support for the review | 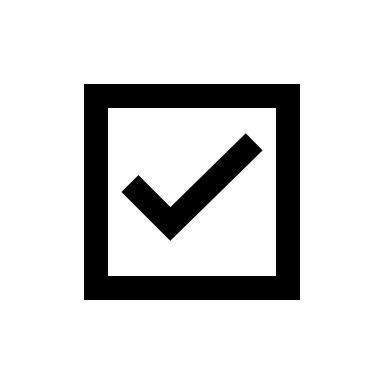 |  | Pg 11 lines 4-5. |
| Sponsor | 5b | Provide name for the review funder and/or sponsor |  | 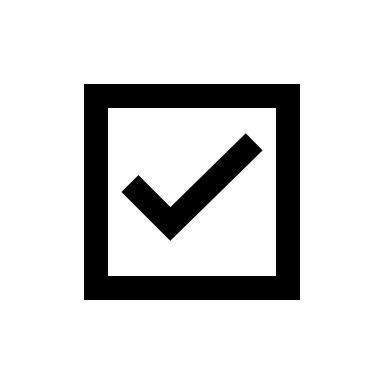 | Not applicable (no funder/sponsor) |
| Role of sponsor/funder | 5c | Describe roles of funder(s), sponsor(s), and/or institution(s), if any, in developing the protocol |  | 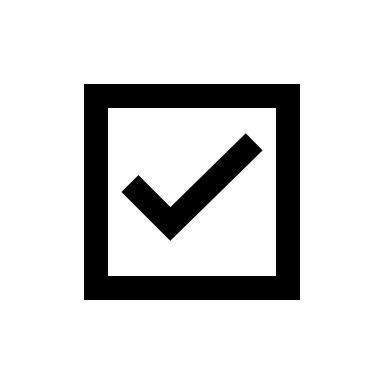 | Not applicable (no funder/sponsor) |
| **INTRODUCTION** | | | | | |
| **Rationale** | 6 | Describe the rationale for the review in the context of what is already known | 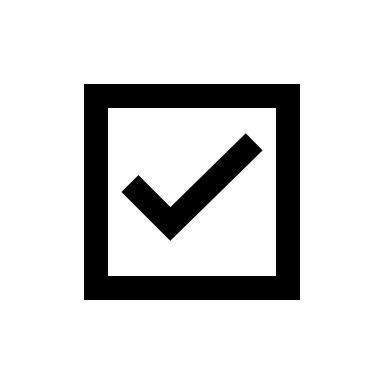 |  | Pg 2, Lines 14-Pg 3 line 25 |
| **Objectives** | 7 | Provide an explicit statement of the question(s) the review will address with reference to participants, interventions, comparators, and outcomes (PICO) | 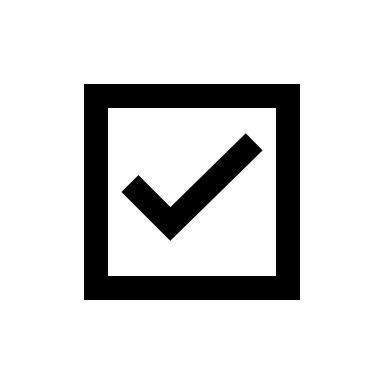 |  | Pg 3 line 18-25 |
| **METHODS** | | | | | |
| **Eligibility criteria** | 8 | Specify the study characteristics (e.g., PICO, study design, setting, time frame) and report characteristics (e.g., years considered, language, publication status) to be used as criteria for eligibility for the review | 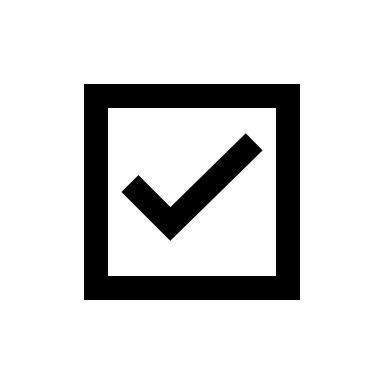 |  | Pg 4 lines 21-36 and Table 1 |
| **Information sources** | 9 | Describe all intended information sources (e.g., electronic databases, contact with study authors, trial registers, or other grey literature sources) with planned dates of coverage | 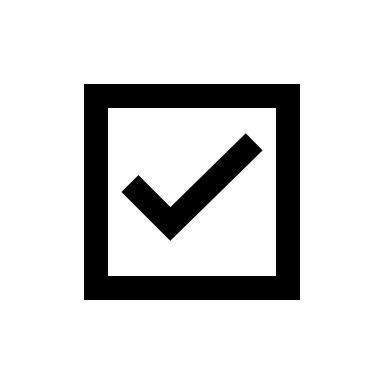 |  | Pg 7, lines 2-19 |
| **Search strategy** | 10 | Present draft of search strategy to be used for at least one electronic database, including planned limits, such that it could be repeated | 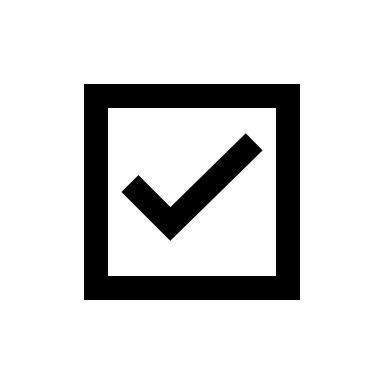 |  | Pg 6 lines 3-10 and Table 2 |
| ***STUDY RECORDS*** | | | | | |
| Data management | 11a | Describe the mechanism(s) that will be used to manage records and data throughout the review | 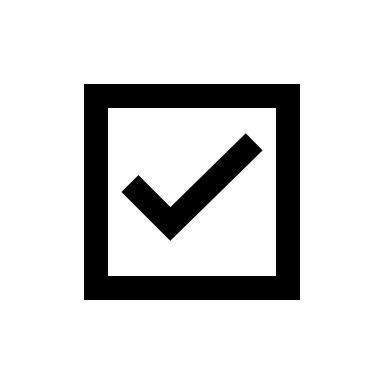 |  | Pg 8 lines 2-3 |
| Selection process | 11b | State the process that will be used for selecting studies (e.g., two independent reviewers) through each phase of the review (i.e., screening, eligibility, and inclusion in meta-analysis) | 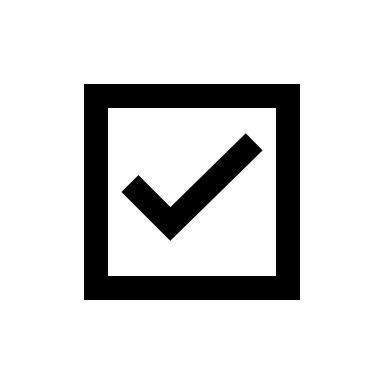 |  | Pg 8 lines 1-20 |
| Data collection process | 11c | Describe planned method of extracting data from reports (e.g., piloting forms, done independently, in duplicate), any processes for obtaining and confirming data from investigators | 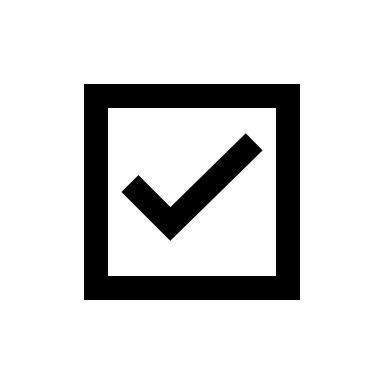 |  | Page 8 line 21-35 |
| **Data items** | 12 | List and define all variables for which data will be sought (e.g., PICO items, funding sources), any pre-planned data assumptions and simplifications | 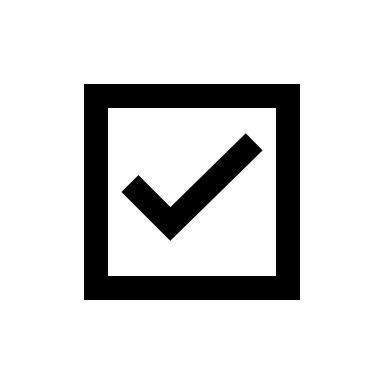 |  | Pg 9, line 1-20 and Appendix II |
| **Outcomes and prioritization** | 13 | List and define all outcomes for which data will be sought, including prioritization of main and additional outcomes, with rationale | 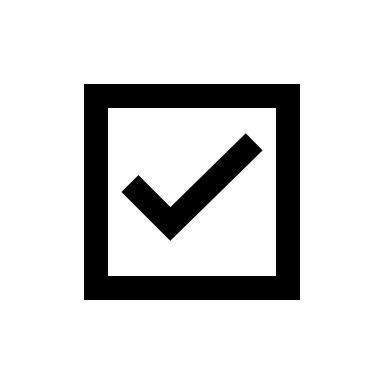 |  | Pg 9, line 21-31 |
| **Risk of bias in individual studies** | 14 | Describe anticipated methods for assessing risk of bias of individual studies, including whether this will be done at the outcome or study level, or both; state how this information will be used in data synthesis | 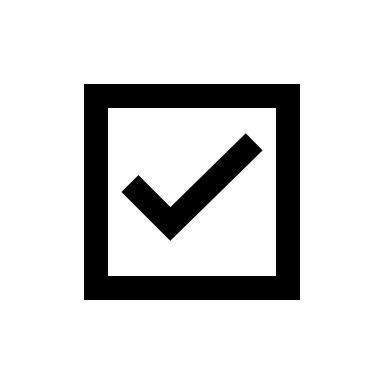 |  | Pg 9 line 42- Pg 10 line 7 |
| ***DATA*** | | | | | |
| **Synthesis** | 15a | Describe criteria under which study data will be quantitatively synthesized | 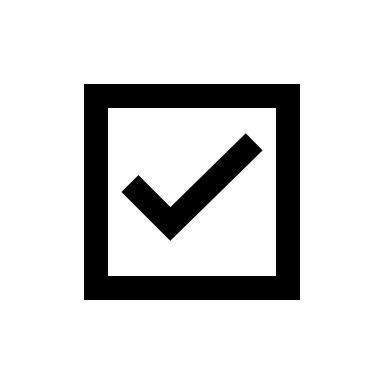 |  | Pg 9 line 4-10 |
|  | 15b | If data are appropriate for quantitative synthesis, describe planned summary measures, methods of handling data, and methods of combining data from studies, including any planned exploration of consistency (e.g., *I* ^2^, Kendall’s tau) | 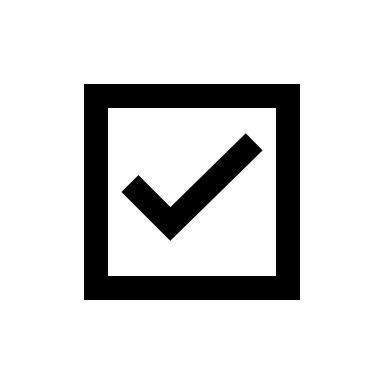 |  | Pg 9 line 4-10 |
|  | 15c | Describe any proposed additional analyses (e.g., sensitivity or subgroup analyses, meta-regression) |  | 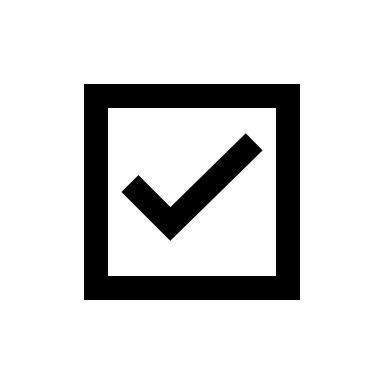 | Not applicable –no inferential statistics or subgroup analysis required |
|  | 15d | If quantitative synthesis is not appropriate, describe the type of summary planned | 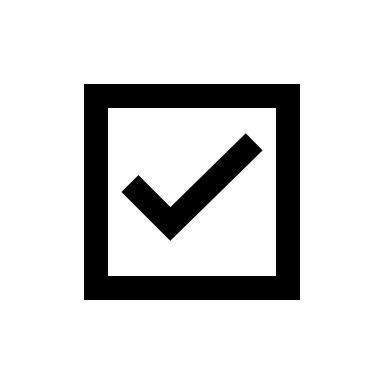 |  | Pg 9, lines 11-20 – Thematic qualitative analysis |
| **Meta-bias(es)** | 16 | Specify any planned assessment of meta-bias(es) (e.g., publication bias across studies, selective reporting within studies) |  | 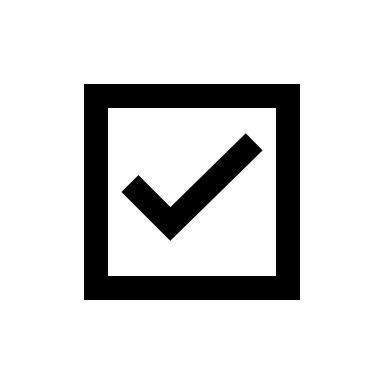 | Not applicable |
| **Confidence in cumulative evidence** | 17 | Describe how the strength of the body of evidence will be assessed (e.g., GRADE) | 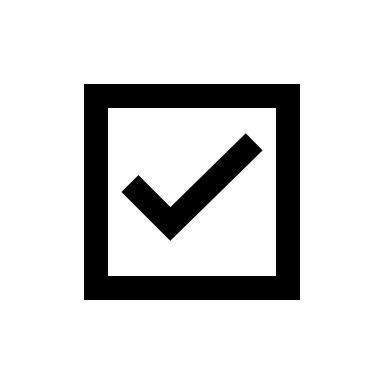 |  | No formal quantification of this will be done as scoping reviews are inclusive of all articles. However, in completed review, qualitative commentary will allude to the diversity of article types and richness of data therein. |
