# Supplementary material for: Beyond the bedside: protocol for a scoping review exploring the experiences of non-practicing healthcare professionals within health professions education
Source: Syst Rev. 2023 Nov 9;12:207. doi: 10.1186/s13643-023-02364-5 (PMC10633985; doi:10.1186/s13643-023-02364-5)
Supplement: Supplementary file 2 — Additional file 2. Data extraction form draft. [file 13643_2023_2364_MOESM2_ESM.docx]

# Scoping Review Extraction Template

**General information**

**Title:**

**Lead author (surname, first name):**

**Year of publication:**

**Country in which the study conducted (Select one from list below):**

1. Australia

2. Canada

3. New Zealand

4. United States

5. UK

6. Other…

**Characteristics of included studies**

**Type of article (Select one from list below):**

1. Research Paper - quantitative
2. Research Paper - qualitative
3. Research Paper - mixed methods
4. Literature review
5. Commentary
6. Letter to editor
7. Thesis/dissertation
8. Abstract (e.g., for conference)
9. Other…

**If research study....**

**Methods (Select one from list below):**

1. NOT RESEARCH STUDY

2. Questionnaire/Survey

3. Interviews (semi-structured/structured)

4. Focus Groups

5. Other…

**Total number of participants (if not research study, enter N/A here):**

**Healthcare Professional Group(s) being discussed in article (Select as many as applicable from list below):**

1. Dentists

2. Doctors

3. Midwives

4. Nurses

5. Occupational therapists

6. Pharmacists

7. Physiotherapists

8. Psychologists

9. Other…

**Thematic/Topic Analysis**

**Inclusion criteria that apply to article (Select as many as applicable from list below):**

1. *REASONS* for *LEAVING* clinical practice

2. *PREVALENCE* of *LEAVING* clinical practice (e.g., by specialty, gender, background, profession...)

3. Non-practicing experience/journey/job

4. *REASONS* for non-practicing healthcare professionals *RETURNING* to clinical practice

5. *PREVALENCE* of *RETURNING* to clinical practice (e.g., by specialty, gender, background, profession...)

6. Other…

**Thematic data extraction**

| **Topic** | **Article (Extract article data that fits the topic(s) below** |
| --- | --- |
| REASONS for LEAVING clinical practice |  |
| PREVALENCE of LEAVING clinical practice (e.g., by specialty, gender, background, profession...) |  |
| Non-practicing job/role |  |
| Non-practicing job location/geography (e.g., hospital, university, community...) |  |
| Non-practicing experience - positives |  |
| Non-practicing job/role - negatives |  |
| Non-practicing job/role - neutrals |  |
| REASONS for non-practicing healthcare professionals RETURNING to clinical practice |  |
| PREVALENCE of RETURNING to clinical practice (e.g., by specialty, gender, background, profession...) |  |
| Licensing/practice restrictions/permissions |  |
| Other |  |

**Link to spreadsheet for backward citation http:XXX (REDACTED FOR PUBLICATION PURPOSES)**
